# ALKBH10B mediated epitranscriptomic regulation enhances drought tolerance of *Arabidopsis thaliana* via hormonal cross-talk and sustained photosynthesis

**DOI:** 10.1007/s00299-026-03951-1

**Published:** 2026-08-21

**Authors:** Yasira Shoaib, Rongpeng Han, Sabarna Bhattacharyya, Alexandra Finkenauer, Katharina Gutbrod, Peter Dörmann, Hunseung Kang, Ute C. Vothknecht, Fatima Chigri

**Affiliations:** 1 Institute for Cellular and Molecular Botany (IZMB), University of Bonn, Kirschallee 1, 53115 Bonn, Germany; 2 Department of Applied Biology, College of Agriculture and Life Sciences, Chonnam National University, Gwangju, 61186 Korea; 3 Department of Plant Genetics, Gembloux Agro-Biotech, Avenue de la Faculte d’Agronomie 2A, 5030 Gembloux, Belgium; 4 Institute of Molecular Physiology and Biotechnology of Plants (IMBIO), University of Bonn, Karlrobert-Kreiten-Straße 13, 53115 Bonn, Germany

**Keywords:** Abiotic stress, Phytohormones, Transcriptomics, Epitranscriptomics (m^6^a), Post-transcriptional modification

## Abstract

**Key message:**

The m⁶A demethylase ALKBH10B emerges as a crucial post-transcriptional regulator of hormonal crosstalk, photosynthetic maintenance, and mitochondrial energy metabolism to fine-tune plant stress adaptation to drought**.**

**Abstract:**

Epitranscriptomic N⁶-methyladenosine (m⁶A) mRNA modification has emerged as an important regulatory layer of gene expression and protein synthesis in plant stress adaptation. In this study, expression profiling revealed that drought stress induces a global reprogramming of the m^6^A machinery in *Arabidopsis thaliana*, with the m^6^A demethylase *ALKBH10B* exhibiting a more pronounced time-dependent response. *ALKBH10B* transcription is induced by ABA, most likely via ABRE and MYB cis-elements, whereas jasmonate alleviates this activation. Moreover, *ALKBH10B* overexpressing plants (OX-1) accumulated wild type (WT) levels of ABA under drought stress but displayed a markedly reduced content in jasmonic acid (JA) and jasmonoyl-isoleucine (JA-Ile). They furthermore exhibit enhanced stomatal closure, improved water-use efficiency, and thus an enhanced drought tolerance. Transcriptome analysis revealed that drought stress induces fewer transcriptional changes in OX-1 plants compared to WT and the drought-sensitive *alkbh10b-1* mutant. Differential expression and GO enrichment analyses highlight that photosynthesis-related processes were most affected by ALKBH10B activity, correlating with a preserved chlorophyll content and PSII efficiency in OX-1. Analysis by m⁶A-IP-qPCR showed that ALKBH10B directly demethylates specific photosynthesis-related and drought-responsive genes, while other transcripts are indirectly regulated by ALKBH10B-dependent processes. Moreover, transcripts associated with respiration were enriched in OX-1, indicating that ALKBH10B also supports mitochondrial energy metabolism during drought. Together, our results suggest that ALKBH10B enhances drought tolerance by coordinating m⁶A-dependent transcriptional and post-transcriptional regulation to maintain photosynthetic capacity and mitochondrial energy metabolism, as well as fine-tuning ABA-jasmonate crosstalk.

**Supplementary Information:**

The online version contains supplementary material available at https://doi.org/10.1007/s00299-026-03951-1.

## Introduction

Plants, being sessile organisms, are subjected to various biotic and abiotic stresses. Drought is considered one of the major abiotic stresses, causing a series of morphological, physiological, biochemical, and molecular changes that affect plant development and crop yield (Lozano-Elena et al. 2022). Thus, with an increasing human population and a decrease in arable land, there is an intense need for the development of drought-resilient crops to maintain adequate food production.

Plants can sense the lack of water early on under progressive drought conditions and trigger complex signalling cascades, which ultimately regulate molecular processes including gene expression to facilitate an appropriate response (Osakabe et al. 2014; Nakashima et al. 2014). The aim is to prevent water loss, e.g., by stomata closure, adjustment of water-utilizing processes, such as photosynthesis, and/or initiation of escape mechanisms, such as early flowering (Haghpanah et al. 2024). Various physiological and molecular analyses, not only in Arabidopsis but also many other plants, have identified phytohormones as key signalling molecules that regulate drought stress responses (Aimar et al. 2011; Gupta et al. 2020). Among these, ABA has been shown to play a critical role in drought tolerance both through direct regulation of stomatal closure as well as regulating production of various drought-responsive proteins (Nakashima et al. 2014; Chen et al. 2020). Promoter regions of drought-responsive genes often contain two cis-acting elements, namely ABRE (Abscisic Acid Responsive Element) and DRE (Dehydration Responsive Element), to facilitate the drought-stress related regulation of gene expression (Nakashima and Yamaguchi-Shinozaki 2010). However, other phytohormones have also been shown to be involved in the drought response, including jasmonates, especially JA-Ile, the biologically active derivative of JA (Alam et al. 2014; Cheong and Choi 2003; Mahmud et al. 2022). These hormones do not act independently from each other and cross-talk has been described between jasmonate and ABA signalling with regards to drought responses. It includes co-regulation of transcriptional ABA responses by alleviating the ABA-mediated induction of drought-responsive genes, such as *RD29A* in Arabidopsis and *StRD29* in *Solanum tuberosum* (Bleker et al. 2024).

In recent years, it has been discovered that plants experiencing environmental stresses extensively reprogram post-transcriptional gene regulatory processes. Among around 170 known post-transcriptional mRNA modifications (Boccaletto et al. 2018; Xuan et al. 2018) N6-methyladenosine (m^6^A) is the most abundant, dynamic, and reversible internal mRNA modification in eukaryotes (Liu and Pan 2016; Arribas-Hernandez and Brodersen 2020). The role of m^6^A has been explored in various abiotic stress responses, such as salinity, drought, and cold stress (Lu et al. 2020; Hu et al. 2019; Zheng et al. 2021; Zhang et al. 2021; Shoaib et al. 2021; Vicente et al. 2023; Pan et al. 2024; Cai et al. 2024). m^6^A is installed by methyltransferases known as writers, removed by demethylases called erasers, and read by RNA-binding proteins called readers (Hu et al. 2019). In plants, an m^6^A writer complex has been identified whose core components include METHYLTRANSFERASE A and B (MTA, MTB), the adapter protein VIRILIZER (VIR), FKBP12-INTERACTING PROTEIN 37 (FIP37), and a ubiquitin E3 ligase called HAKAI (Zhong et al. 2008; Shen et al. 2016; Zhang et al. 2019; Wang et al. 2022; Xu et al. 2022). Recently, MTA has been shown to contribute to drought stress resistance in Arabidopsis, apple, and poplar (Lu et al. 2020; Hou et al. 2022; Ganguly et al. 2025). Notable reader proteins that recognize m^6^A modification belong to YTH domain proteins and are named Evolutionary Conserved C-Terminal Region (ECT) (Reichel et al. 2019). FLK is another reader protein belonging to the KH domain family (Fabian et al. 2023). These reader proteins decode the m^6^A modification and mediate the downstream processes, such as splicing, alternative polyadenylation, RNA export, stability and translation (Hu et al. 2019).

Demethylases involved in m^6^A mRNA modification belong to the family of ALKYLATION B HOMOLOG proteins (ALKBH), whose members are involved in various cellular processes related to DNA repair and gene regulation. Among the 14 ALKBH proteins in Arabidopsis, ALKBH9B, ALKBH9C and ALKBH10B are well-characterized m^6^A eraser (Martinez-Perez et al. 2021; Duan et al. 2017; Amara et al. 2022). ALKBH9B is important in viral defence and also involved in promoting the mobilization of heat-activated long terminal repeat retrotransposons (Martinez-Perez et al. 2021; Fan et al. 2023). ALKBH9C is crucial for seed germination and seedling growth under salt, osmotic stress and ABA by affecting the stability of various stress-responsive transcripts (Amara et al. 2022). ALKBH10B plays an important role in floral transition by demethylation of transcripts of flowering-related genes, such as *FLOWERING LOCUS T* and *SQUAMOSA PROMOTER BINDING PROTEIN-LIKE 3* and* 9* (Duan et al. 2017). Moreover, ALKBH10B also affects the expression of various genes involved in salt, osmotic stress, or ABA responses (Shoaib et al. 2021; Tang et al. 2021; Han et al. 2023).

While recently shown to positively regulate drought resistance in Arabidopsis (Han et al. 2023), the exact contribution of ALKBH10B to drought tolerance is not well defined. Here, we combine physiological analyses, hormone quantification, transcriptomics, and m⁶A profiling to elucidate the molecular mechanisms by which ALKBH10B contributes to drought tolerance. We show that constitutive increased expression of *ALKBH10B* in OX-1 plants leads to improved water retention, reduced jasmonate accumulation, and maintenance of photosynthetic efficiency compared to WT and the *alkbh10b-1* null-mutant despite comparable ABA levels across genotypes. RNA-seq analysis showed large alteration in the transcriptional landscape of the different lines, affecting expression of genes related to photosynthesis and drought response. Analysis by m^6^A-IP-qPCR identified direct alteration in m^6^A enrichment in some of these genes in OX-1 and *alkbh10b-1*. However, it also manifests that changes in transcript levels of many genes seem rather indirectly affected by *ALKBH10B*. RNA-seq analysis also revealed that genes associated with mitochondrial respiration and ATP synthesis were enriched in OX-1, indicating that increased removal of m^6^A mRNA modification by ALKBH10B can support energy metabolism during drought. Collectively, our findings demonstrate that ALKBH10B-driven mRNA demethylation regulates hormonal crosstalk, photosynthetic maintenance, and mitochondrial energy metabolism and establishes ALKBH10B as a key epitranscriptomic regulator in drought response that fine-tunes plant stress adaptation by coordinating transcriptional regulation and hormone balance.

## Materials and methods

### Plant materials and growth conditions

In this study, we used *Arabidopsis thaliana* Columbia ecotype (Col-0), the T-DNA insertion line *alkbh10b* (Salk_004215c, Fig. S1A) and two over-expression lines (OX-1 and OX-2) expressing an *ALKBH10B-FLAG* fusion under the control of CaMV 35S promoter (Han et al. 2023). The *aba2-1* and *jar1-11* mutant lines were previously described (Cheng et al. 2002; Mahmud et al. 2022). *Nicotiana benthamiana* was used for transient infiltration. For drought stress, Arabidopsis seeds were sown directly in potting soil whereas for other experiments, they were surface sterilized with 70% (v/v) ethanol for 3 min, 6% (v/v) sodium hypochlorite (NaOCl) for 5 min and then five times rinsed with distilled water and sown on ½ MS (Duchefa Biochemie, The Netherlands) plates containing 1% (w/v) sucrose and 0.4% (w/v) phytoagar. The plates were maintained in the dark at 4 °C for three days for vernalization before being transferred to the growth chamber. To study the regulation by hormones, Arabidopsis seedlings were grown for three weeks on ½ MS plates and treated either with 50 µM (+)-ABA, 50 µM MeJA (SERVA, Germany), or 50 µM ABA + 50 µM MeJA or with the same volume of H_2_O as a mock treatment. Plants were harvested after 3-, 6-, and 24-h treatment, frozen in liquid nitrogen, and RNA was extracted as described below. All plants were grown at 22±2 °C in a climate-controlled growth chamber under long-day conditions (16 h light/8 h dark) with a light intensity of 100 μmol photon*m^−2^*s^−1^ (Philips TLD 18 W lamps of alternating 830/840 light temperature).

### Phenotyping under drought stress, measurement of relative leaf water content, and stomatal aperture and density

Seven-day-old seedlings of WT, *alkbh10b-1*, and OX-1 grown on soil were transferred to individual pots containing an equal amount of soil. Plants were watered regularly until they were 18 days old and drought stress was applied by withholding water. After 10–12 days plants were rewatered for three days, and the survival rate was measured. The survival percentage was calculated as (number of survived plants/total number of plants)*100.

The relative leaf water content (RWC) was quantified and calculated as previously described (Mahmud et al. 2022). In general, leaves no. 5–8 were used from 5 individual plants for each genotype. For the determination of stomatal aperture, plants from WT, *alkbh10b-1* mutant, and OX-1 lines grown under normal conditions (control) were incubated in imaging buffer (10 mM MES, 5 mM KCl, 50 μM CaCl_2_, pH 6.15) for two hours. The lower epidermis from the 6th & 7th leaves was carefully peeled, separated from mesophyll cells, and depicted using bright fields in a Confocal Laser Scanning Fluorescence Microscope (Leica SP8 Lightning, Leica, Germany) using the internal LAS X software. Stomatal aperture was measured using ImageJ software (Schindelin et al. 2012). Stomatal density was determined as described in (Bhattacharyya et al. 2025) using data from 6 individual plants for each genotype.

### Measurement of chlorophyll content, photosystem II activity and stomatal conductance

The chlorophyll content and photosystem II activity were measured in control and drought-exposed plants. Chlorophyll was measured through a DUALEX optical leafclip meter (Pessl Instruments, Austria). For the measurement of stomatal transpiration and photosystem II activity, a LI-COR (LI-6000) porometer/fluorometer (LI-COR Environmental GmbH, Germany) was used. All measurements were performed on 2 leaves per plant and 10 independent plants from each line were used.

### Promoter analysis and molecular cloning in pBIN19-ANX vector

The 1-kb promoter region upstream of the *ALKBH10B* transcription initiation site was analysed for cis-acting elements through PlantCARE (https://bioinformatics.psb.ugent.be/ webtools/plantcare/html/). To measure the promoter activity under different hormone treatments, the 1 kb promoter region of *ALKBH10B* (p*ALKBH10B*) was amplified with forward and reverse primers containing Apa1 and Kpn1 restriction sites, respectively, using gDNA as a template and ligated into a pBIN19 vector containing the firefly luciferase (FLUC) reporter gene. The construct cassette is shown in Fig S1D, and the primer sequences are listed in Supplementary Table S1. After confirming the correct sequence, the vector containing p*ALKBH10B*::fluc was transformed into Agrobacterium strain GV3101.

### Transient transformation of *Nicotiana benthamiana* and luciferase assays

The transient infiltration and luciferase-based assays were performed as described previously (Bleker et al. 2024) with some slight modifications. Briefly, Agrobacterium cells carrying the pBIN-p*ALKBH10B*::fluc construct were grown overnight at 28 °C in YEB medium containing 100 µg/ml rifampicin, 25 µg/ml kanamycin and 1 M MgCl_2_. The culture was then centrifuged at 3500 rpm for 5 min, resuspended in infiltration medium (20 mM citric acid, 2% (w/v) sucrose) and adjusted to OD_600_ = 0.5 for infiltration into the leaf number 6 and 7 of four-week-old *Nicotiana benthamiana* plants. Only healthy plants without visible signs of pathogen infection or tissue damage were used for infiltration and subsequent luciferase measurements. Three days after infiltration, leaf discs of identical size (ø 6 mm) were excised from the infiltrated regions and immersed in autoclaved water containing 30 µM D-luciferin together with either 50 µM ABA, 50 µM MeJA, 50 µM ABA+ 50 µM MeJA or 50 µM ABA+25 µM MeJA and H_2_O as mock control in a 96 well plate. FLUC luminescence was recorded in a Multi-Mode Microplate reader (Tristar^2^, Berthold GmbH, Germany) after three hours. During the measurement the leaf discs were kept in darkness. The experiment was repeated independently three times each with four technical replicates.

### RNA extraction, cDNA synthesis and RT-qPCR

Total RNA of four-week-old plants grown under control and drought stress conditions was extracted using the Genematrix Universal RNA Purification Kit (Roboklon, Germany) according to the manufacturer’s instructions. RNA integrity was assessed on a 1% agarose gel and the concentration was measured with a Nanodrop photometer (Nanodrop™ One, Thermo Fisher Scientific). cDNA was synthesized from 1.5 µg of total RNA with oligodT-_18_ and random hexamer primers following the manufacturer’s instructions of the RevertAid First Strand cDNA Synthesis Kit (Thermo Fisher Scientific, Germany). RT-qPCR was performed in a 96-wells plate using the CFX96 real-time thermal cycler system (Bio-Rad, Germany) and SYBR Green PCR master mix (Thermo Fisher Scientific, USA). All transcript levels were normalized against the geometric means of two reference genes, namely *ACTIN2* and *TUBULIN 2,* and the relative expression level was quantified using the 2^-^ΔΔCT^ method. All primers are listed in Supplementary Table S1.

### Measurement of phytohormones

Whole rosette leaves of four-week-old plants grown under control and drought stress conditions, two plants for each sample, were frozen in liquid nitrogen and ground in a pre-cooled mortar with a pestle. Extraction and quantification of hormones were done as previously described (Pan et al. 2010). To 50 mg of the plant powder, 25 µl of internal standards was added. The internal standards used were d_6_-ABA (OlChemIm, Olomouc, Czech Republic) for ABA quantification, d_6_-SA (CamPro Scientific, Berlin, Germany) for SA quantification, and H_2_JA (Sigma-Aldrich, St. Louis, MO, USA) for JA and JA-Ile quantification. The hormones were extracted with 500 µl of extraction solvent consisting of 2-propanol/H_2_O/conc. HCl (2:1:0.002, v/v/v) and shaken at 100 rpm for 30 min at 4 °C. To each sample, 1 ml of dichloromethane was added and the samples were shaken for 30 min at 4 °C. The samples were then centrifuged at 13,000 × g for 5 min at 4 °C to achieve phase separation. From the lower phase, 900 µl were transferred to a screw-cap vial and the sample was dried completely using a nitrogen evaporator. Residual tissues were resuspended in 0.1 ml methanol: 0.1% formic acid in water (1:1, v/v). An aliquot of 50 µl of the sample solution was injected onto the reverse-phase C18 Gemini HPLC column and analysed using a QTRAP 6500+ LC–MS/MS system (Sciex, Germany). The concentrations of ABA, JA, JA-Ile and SA (salicylic acid) were quantified relative to the internal standards and expressed as ng/g F.W.

### RNA-sequencing and data analysis

RNA sequencing was performed using three biological replicates, each consisting of pooled RNA extracted from the rosette leaves of two individual plants grown for 4 weeks under well-watered and drought stress conditions using the Direct-zol RNA Purification kit (Zymo Research Corporation, U.S.A.). An RNA quality check was carried out using a Tapestation 4200 (Agilent, U.S.A.). Approximately 100 ng of RNA was used for library preparation and 3’ mRNA sequencing was done by the NGS Core Facility (Medical Faculty at the University of Bonn, Germany) using a NOVASEQ6000 (Illumina, USA) sequencer. An average sequencing depth of ~10 million raw reads per sample (Supplementary Table S2) was generated. The FASTQ raw files were processed to remove low-quality bases and adapter sequences through the Trimmomatic method (Bolger et al. 2014). To confirm the removal of adapter sequence after trimmomatic processing, FastQC was done on a Linux system. The reads were aligned through HiSAT2 software against the concatenated reference genome of Arabidopsis (http://plants.ensembl.org/info/data/ftp/index.html). The Gene Counts were generated from the aligned BAM files through the Feature Counts function in RStudio (Liao et al. 2014). The differential gene expression was carried out using EdgeR. The *p-*values were corrected using the False Discovery Rate (FDR) method (Benjamini and Hochberg 1995) and subsequently the FDR and |Log_2_FC| cut offs were set to 0.05 and 1, respectively.

Volcano plots were generated through the SRplot web tool (Tang et al. 2023) whereas the Principal Component Analysis (PCA) plot and clusters were generated and visualized with the iDep2 web tool (Ge et al. 2018). Genes selected for comparative transcriptomic analysis were grouped into four clusters using K-means clustering based on their drought-responsive Log₂FC values in WT, *alkbh10b-1*, and OX-1. Within iDep2 row-wise Z-score normalization was applied to the Log_2_FC values for heatmap visualisation, allowing comparison of relative expression patterns across the three genotypes. Gene Ontology (GO) analyses of the obtained DEGs were performed using ShinyGO (Ge et al. 2020).

### m^6^A immunoprecipitation-qPCR

The m^6^A- immunoprecipitation (IP)-qPCR was performed as described previously (Hu et al. 2021). Briefly, mRNA was extracted from total RNA using oligo-d(T)_25_ magnetic beads (New England Biolabs, U.S.A.) and 5 µg was fragmented into around 300-nucleotide-long mRNA fragments by incubation in RNA fragmentation buffer (100 mM Tris–HCl, pH=7.0, 100 mM ZnCl_2_). The fragmented mRNA was mixed with an m^6^A-specific antibody (Synaptic Systems, Germany), and the bound RNAs were eluted. The input and eluted mRNA were reverse transcribed using reverse transcriptase with random hexamer primers and the level of each transcript was measured by RT-qPCR conducted on a Rotor-Gene Q thermal cycle (Qiagen, Germany) using gene-specific primers listed in Supplementary Table S1. The level of m^6^A enrichment of target genes was quantified based on the PCR cycle threshold. The value of the immunoprecipitated sample was normalised against that of the input. Values are the means ± SE of three biological replicates.

## Results

### Drought stress induces changes in the expression level of m^6^A regulatory components

**Fig. 1:**
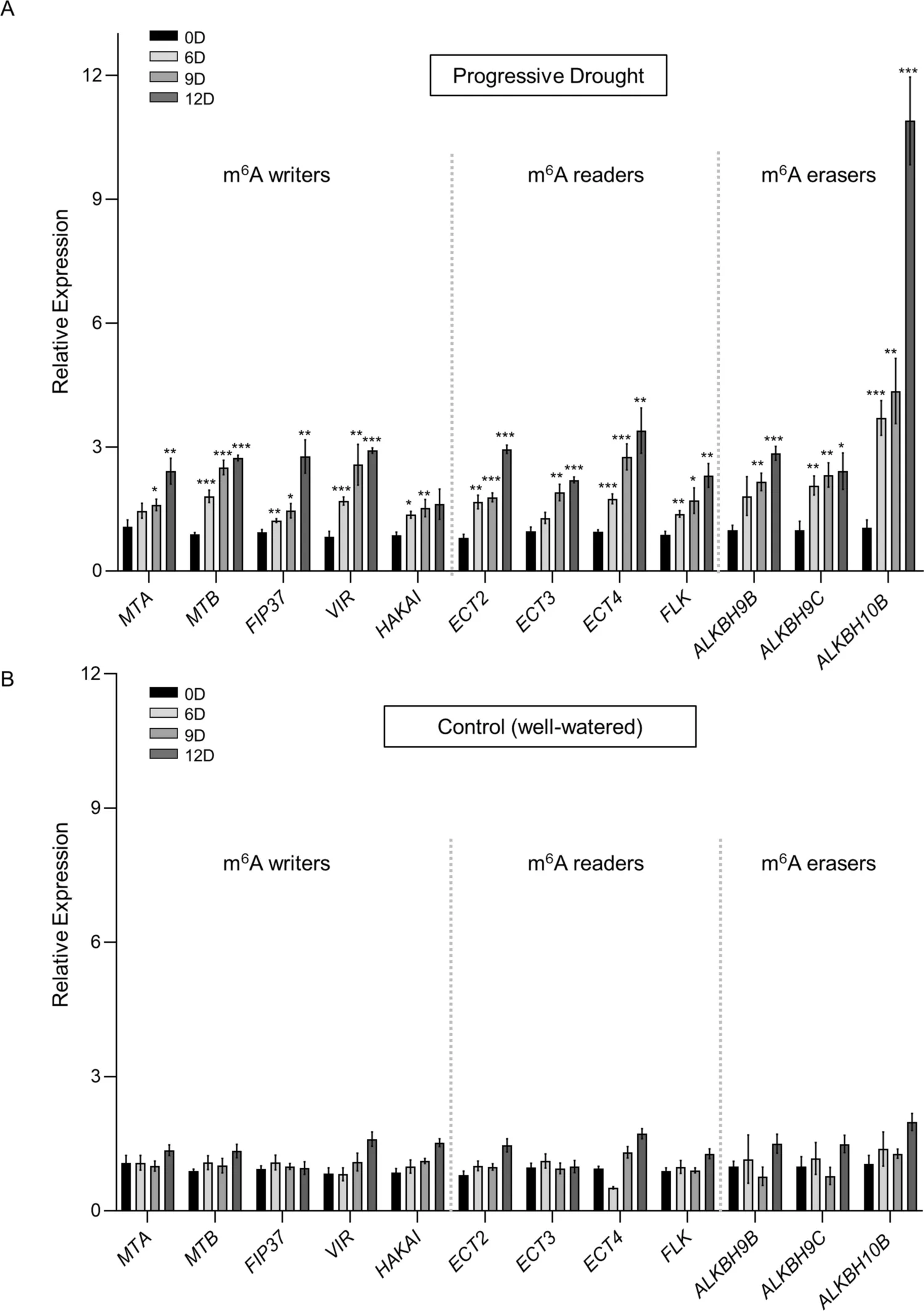
Expression of m^6^A regulatory components under drought stress. The relative expression level of **m**^**6**^**A** writers, readers and erasers was analysed in 18-day-old WT plants grown on soil and exposed to different time periods of **A** progressive drought stress and **B** well-watered conditions. The relative expression was analysed through RT-qPCR after 0, 6, 9, and 12 days of treatment. Values are the mean ± SE of at least three independent biological replicates (*n*=3). Data were analysed through two-tailed student’s t- test (*P*<0.05)

To explore the role of m^6^A mRNA modification in the drought stress response, we quantified the expression of established m^6^A regulatory genes using RT-qPCR. Transcript levels were analysed in rosette leaves of Arabidopsis WT at different time periods of progressive drought treatment and compared to well-watered control conditions (Fig. 1). While drought stress induced a coordinated upregulation across the m^6^A regulatory machinery, the expression kinetics of *ALKBH10B* were particularly noteworthy. Unlike other components, such as the m^6^A writers *MTA*, *MTB*, *FIP37*, *VIR* and *HAKAI*, the m^6^A readers *ECT2*, *ECT3*, *ECT4,* and *FLK*, and the m^6^A erasers *ALKBH9B* and *ALKBH9C*, that exhibited a moderate induction or reached an early plateau, the m^6^A eraser *ALKBH10B* showed a pronounced, time-dependent increased expression, with transcript levels rising substantially between day 6 and day 12 of withholding water (Fig. 1A). Under well-watered conditions, only minor changes in the expression of all these genes occurred (Fig. 1B), suggesting that the drought-induced expression of the m^6^A regulatory genes is not due to developmental effects. Collectively, these results indicate that drought stress induces coordinated reprogramming of the whole m^6^A regulatory machinery, with ALKBH10B emerging as one of the most strongly induced components, particularly at later stages of drought. This unique temporal profile suggests that ALKBH10B may play a specialized role in the transition from early stress signalling to prolonged drought adaptation. We, therefore, focused subsequent analyses on ALKBH10B as a key candidate for mediating m^6^A-dependent responses to drought stress.

### *ALKBH10B* confers drought stress tolerance and regulates water loss through modulation of stomatal aperture

**Fig. 2:**
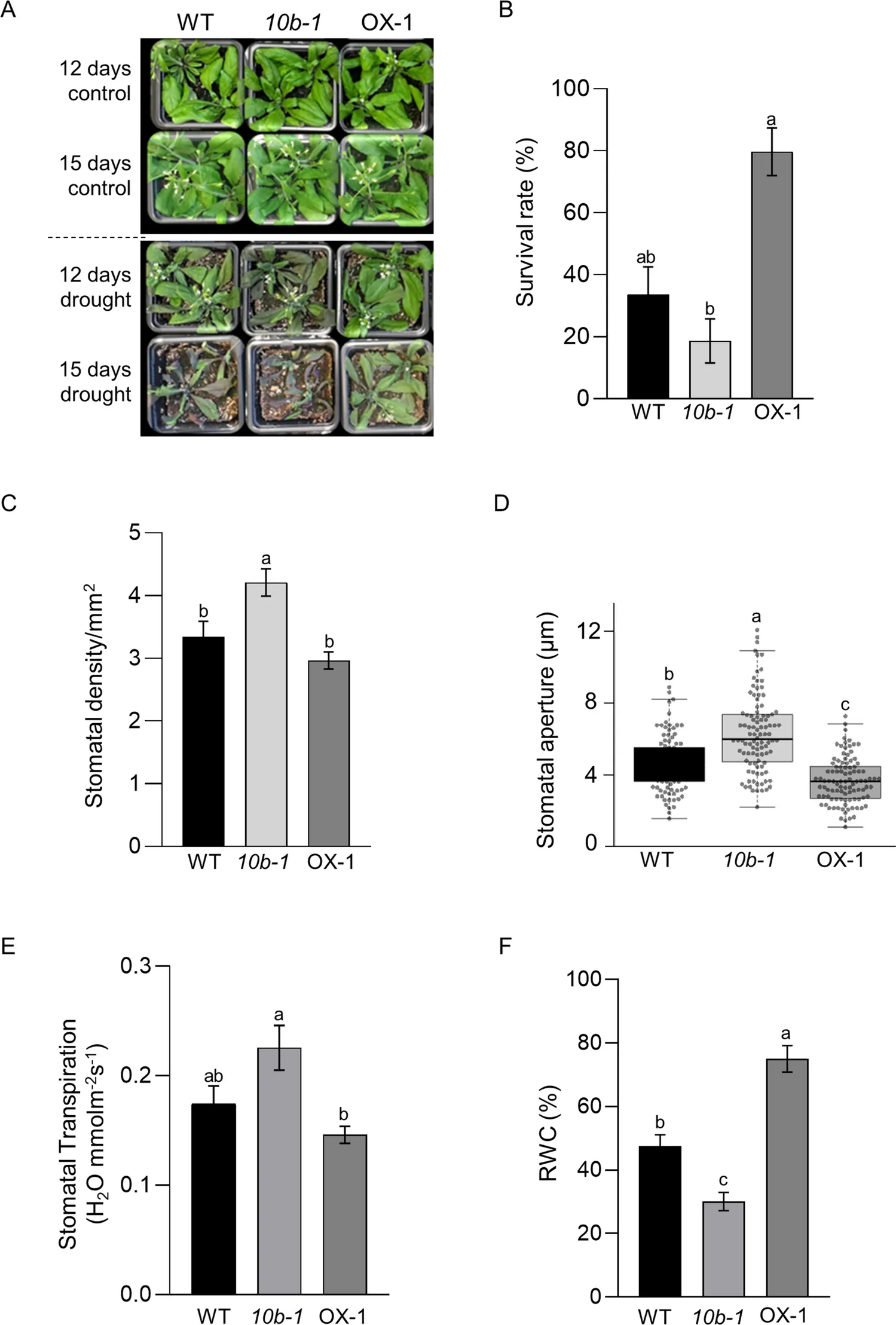
*ALKBH10B* is a positive regulator of drought stress. **A** Phenotypes of WT, *alkbh10b-1*, and OX-1 plants under well-watered and drought stress conditions applied to 18-day-old plants. **B** Survival rate of the plants measured after three days of rewatering. Values are the mean ± SE of three independent biological replicates. **C** Representative pictures showing the stomatal aperture, **D** density and **E** transpiration in leaf (no. 6, 7 and 8) of five individual 28-day-old plants under control conditions. Values are the mean ± SE of *n*=100 for aperture, and *n*=29 for density. **F** Leaf relative water content (RWC) calculated after 12 days of drought stress using three biological replicates each consisting of two plants (*n*=3). All data were analysed through one-way ANOVA and Tukey’s Post-Hoc HSD tests (*P*<0.05)

To further investigate the role of *ALKBH10B* we used the Arabidopsis T-DNA insertion mutant *alkbh10b-1* (SALK_004215C, Fig. S1A) and *35S::ALKBH10B* transgenic plants (OX-1), which were reported in previous studies as knockout and overexpression lines, respectively (Duan et al. 2017; Shoaib et al. 2021; Han et al. 2023). RT-qPCR corroborated a complete loss of *ALKBH10B* transcript in the *alkbh10b-1* mutant and an increased expression in the OX-1 line compared to WT (Fig. S1B). When progressive drought was applied to 18-day-old WT, *alkbh10b-1*, and OX-1 plants by withholding water, *alkbh10b-1* plants showed clear symptoms of drought stress on day 12, while WT showed mild symptoms and OX-1 looked still green. On day 15, both *alkbh10b-1* and WT were severely wilted, whereas the OX-1 plants still showed only slight wilting (Fig. 2A). Consequently, after three days of rewatering, the survival rate of OX-1 plants was approximately 80%, whereas survival rate of WT and *alkbh10b-1* plants was only about 33% and 19%, respectively (Fig. 2B and S2A). The contrasting phenotype of null mutant and overexpression was confirmed using a second OX line (Fig. S2A, OX-2). These results confirm that *ALKBH10B* plays a role as a positive regulator of drought tolerance.

Reduced water loss through transpiration is a key determinant for drought tolerance. Stomatal density was modestly increased only in *alkbh10b-1* but not in WT or the OX lines (Fig. 2C, Fig. S2B). Stomatal aperture measurements, however, revealed wider apertures in *alkbh10b-1* and reduced apertures in OX compared to WT (Fig. 2D, Fig. S2C). Although the reduction in stomatal aperture in the OX lines was moderate in magnitude, it was consistent and statistically significant across biological replicates. These parameters likely contribute to reduced water loss through stomatal transpiration as measured in OX-1 (Fig. 2E), resulting in delayed wilting and decreased drought sensitivity. Accordingly, the relative water content (RWC) on day 12 of progressive drought was higher in OX-1 and reduced in *alkbh10b-1* plants (Fig. 2F**).** We, therefore, interpret stomatal regulation as one, albeit not the only, contributing component of ALKBH10B-mediated drought tolerance.

### *ALKBH10B* expression is regulated by ABA and jasmonates

**Fig. 3:**
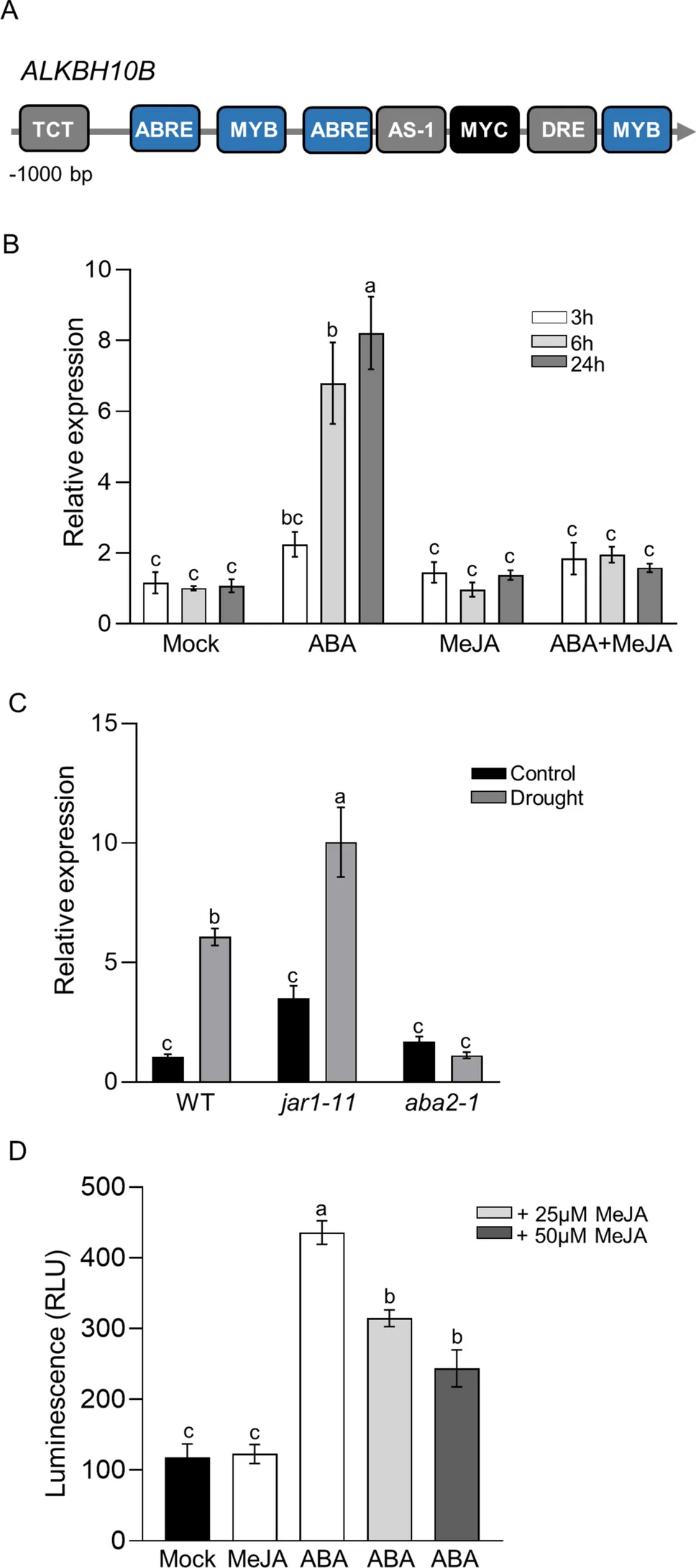
Effect of ABA and MeJA on the expression of *ALKBH10B. A* Analysis of the 1 kb promoter region of *ALKBH10B* showing various cis-regulatory elements responsive to hormones including ABA (ABRE and MYB), JA (MYC), and salicylic acid (AS-1) as well as drought (DRE) and light (TCT) responsive element **B** The relative expression of *ALKBH10B* was quantified in WT plants grown on ½ MS plates and treated for different time points with water (mock), 50 µM ABA, 50 µM MeJA, or 50 µM of each (ABA+MeJA) **C** Relative expression of *ALKBH10B* in WT*, jar1-11,* and *aba2-1* plants grown on soil under control and drought stress conditions **D** Relative luciferase activity measured in leaf discs excised from transiently transformed *Nicotiana benthamiana* leaves carrying the p*ALKBH10B::FLUC* reporter and treated for 3 h with either water (mock), 50 µM ABA, 50 µM MeJA or 50 µM ABA + different concentration of MeJA. Values are the mean ± SE of three independent biological replicates (*n*=3). Data were analysed through two-way ANOVA followed by Tukey’s Post-Hoc HSD tests (*P*<0.05)

Analysis of the 1-kb promoter region of ALKBH10B using the PlantCARE database revealed multiple ABRE (Abscisic Acid Responsive Element) and MYB binding motifs as well as a MYC motif and AS-1 element (Fig. 3A). These elements have been associated with ABA and/or jasmonate-dependent transcription and often act combinatorially in response to different hormones. Further regulatory elements include DRE (dehydration-responsive element), which is associated with ABA-independent stress response and often occurs alongside ABREs. This promoter analysis provides a conceptual framework for understanding hormone-dependent regulation of ALKBH10B.

ABA and jasmonate are important regulators of drought responses (Ullah et al. 2018) and treatment of WT seedlings grown on ½ MS medium confirmed that *ALKBH10B* expression is strongly induced by ABA (Fig. 3B). MeJA application had no influence on *ALKBH10B* expression but co-application of both hormones strongly reduced ABA-mediated *ALKBH10B* induction suggesting an antagonistic interaction. In accordance with these results, drought-induction of *ALKBH10B* was absent in a mutant defective in ABA biosynthesis (*aba2-1)* (Cheng et al. 2002) and enhanced in *jar1-11*, a plant line defective in JA-Ile formation (Mahmud et al. 2022) (Fig. 3C). These data reveal a complex integration of hormonal signals where ABA is a major contributor to drought-induced *ALKBH10B* expression, while jasmonate acts as a negative modulator to calibrate the amplitude of this induction.

Expression of a luciferase reporter driven by the *ALKBH10B* promoter (Fig. S1C). confirmed induction by ABA and attenuation by jasmonate (Fig. 3D) consistent with a regulation mediated by cis-acting promoter elements.

### *ALKBH10B* affects accumulation of ABA and JA

**Fig. 4:**
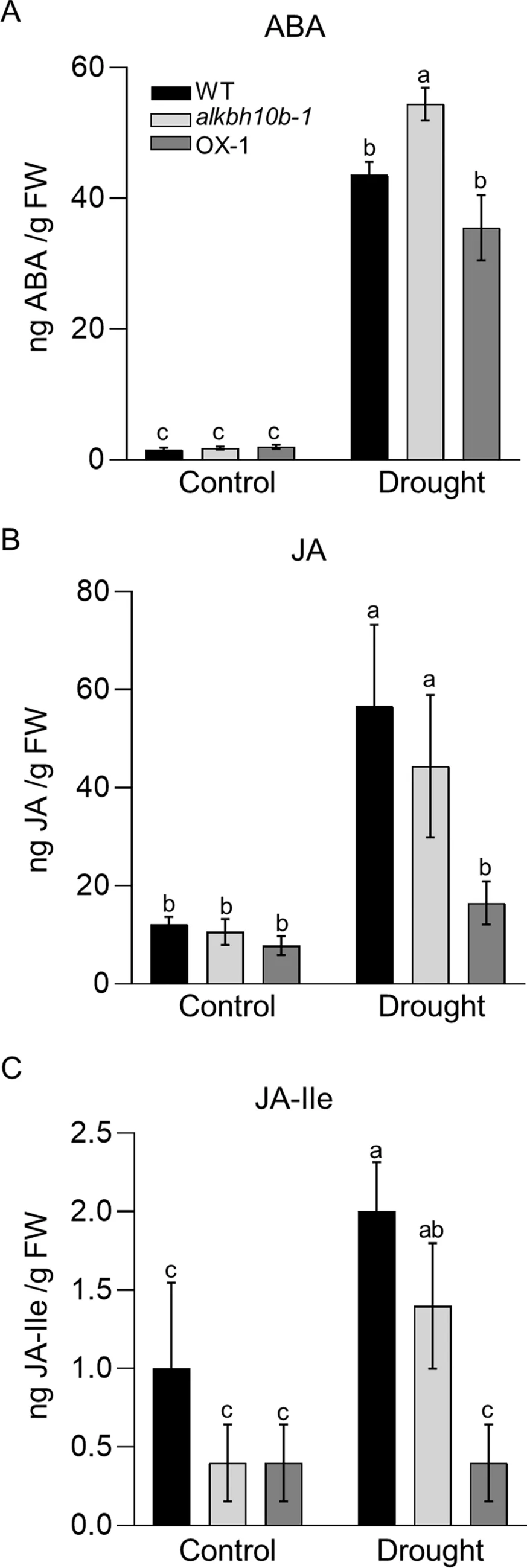
Phytohormone content in WT, *alkbh10b-1* and OX-1 lines. The content of **A** ABA, **B** JA, and **C** JA-Ile was analysed in plants grown on soil under control or 12 days of drought stress conditions**.** Data represent mean ± SE of 5 biological replicates (*n*=5) each containing pooled extracts from 2 plants. Data were analysed through two-way ANOVA followed by Tukey’s Post-Hoc HSD test (*P*< 0.05)

Phytohormones, such as ABA, and jasmonate regulate extensive transcriptional networks, and a subset of these hormone-responsive genes encode proteins that also modulate the hormone’s own levels, thereby establishing feedback loops of hormone content regulation (Zong et al. 2016; Xie et al. 2021; Zhao et al. 2024). We thus examined whether *ALKBH10B* also affects hormonal dynamics under drought stress. To this end, the levels of ABA, JA, and JA-Ile were quantified in WT, *alkbh10b-1* and OX-1 plants under control and drought stress conditions (Fig. 4). Under control conditions, ABA, JA, and JA-Ile levels were low and similar across genotypes (Fig. 4). Under drought, ABA level increased in all genotypes, with a slightly higher accumulation in *alkbh10b-1* mutant (Fig. 4A). By contrast, levels of JA and JA-Ile increased in the WT and the *alkbh10b-1* mutant under drought but remained unchanged in OX-1 (Fig. 4B and C). The reduced jasmonate accumulation in OX-1 may reflect either direct regulation of specific transcripts by ALKBH10B or indirect metabolic consequences of altered transcriptional or physiological states. At present, our data do not allow us to distinguish between these possibilities.

### RNA-seq analysis reveals effect of *ALKBH10B* on respiration and photosynthesis under drought stress

**Fig. 5:**
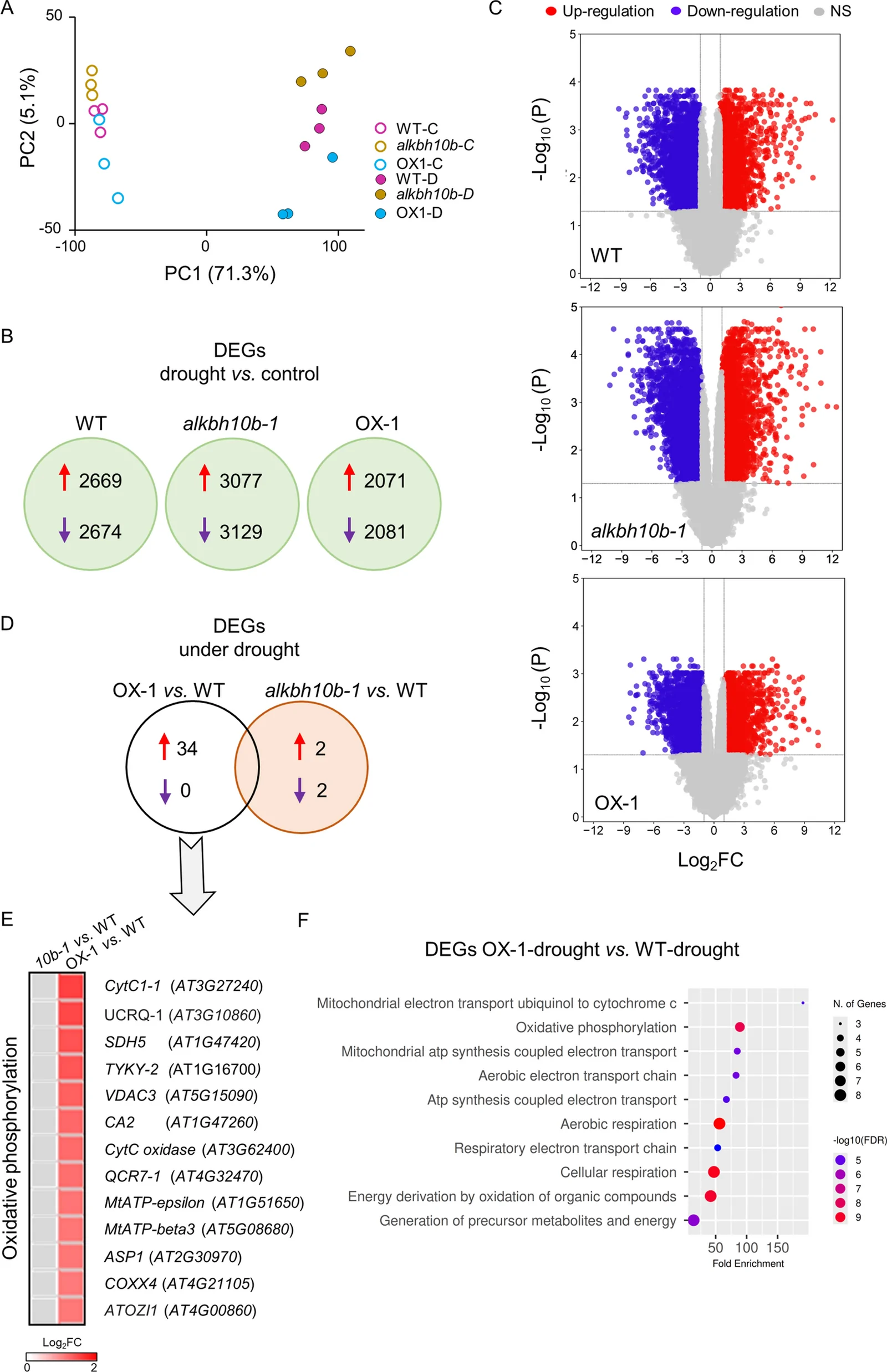
Differentially expressed genes in WT, *alkbh10b-1* and OX-1. **A** Principal component analysis of WT, *alkbh10b-1* and OX-1 samples under control (C) and drought (D) conditions**,** PC1 represents variation based on treatment and PC2 represents variation based on genotype **B** Number of up- and down-regulated genes in WT, *alkbh10b-1* and OX-1 (drought *vs.* control) **C** Volcano plots showing differentially expressed genes (DEGs) in drought *vs.* control samples defined by |Log_2_FC|≥ 1 and FDR<0.05 **D** Number of up- and down-regulated genes in *alkbh10b-1 vs.* WT and in OX-1 *vs*. WT under drought conditions **E** Heatmap and **F** GO biological processes of genes with higher expression levels in OX-1 *vs*. WT under drought conditions. The red squares in the heatmap indicate higher expression levels, whereas grey squares indicate no significant differences in expression

m^6^A mRNA modification can change transcript levels of genes either directly, e.g. by affecting mRNA stability of a specific gene, or indirectly by modulating transcripts of regulatory components/hubs that in turn alter transcription of a larger set of genes. We thus performed RNA-seq analysis on plants of all three genotypes at day 12 of progressive drought compared to well-watered control conditions. The sequencing yielded approximately ~10 million raw reads per sample, with around 85% of the reads successfully aligned to the Arabidopsis reference genome TAIR 10 over all samples (Supplementary Table S2). Principal Component Analysis (PCA) revealed a clear separation of samples based on treatment (PC1, 71.3% variance). The variance among genotypes was much smaller (PC2, 5.1% variance), but the WT was positioned between *alkhb10b-1* and OX-1 under both conditions (Fig. 5A). This indicates that drought stress is the dominant factor shaping the transcriptomic landscape, while genotypic effects are more subtle.

**Fig. 6:**
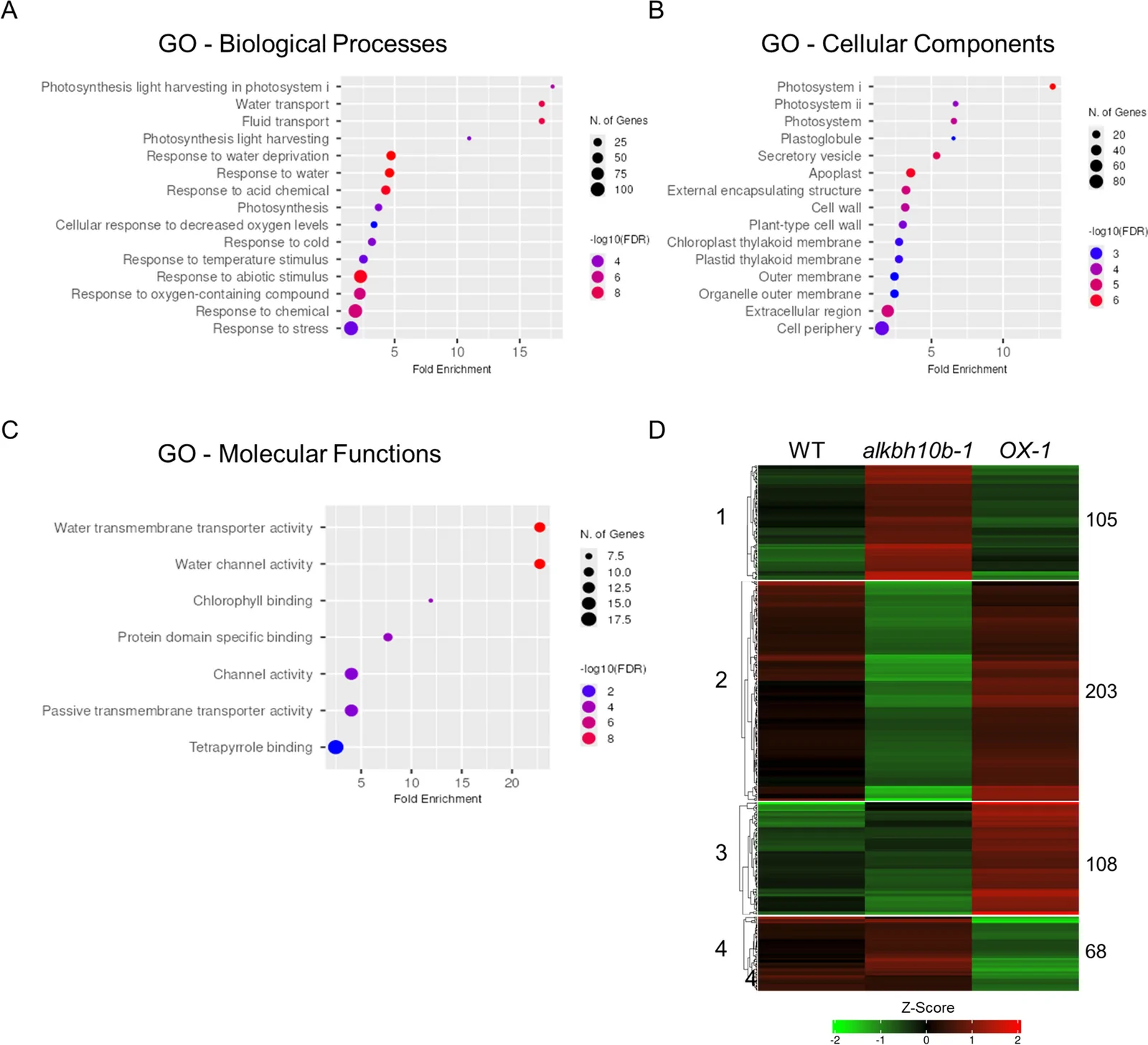
Effect of *ALKBH10B* on transcriptomic variations between drought stress and control conditions. Genes showing differences in drought-responsive expression patterns between *alkbh10b-1* and OX-1 were selected based on differences in drought-induced Log₂FC values (Supplementary Table S4) and used for GO enrichment analyses classified into **A** biological process, **B** cellular components and **C** molecular functions. **D** Heatmap showing the K-means clustering of the selected genes based on their drought-responsive Log₂FC values across WT, *alkbh10b-1*, and OX-1 plants. Four individual expression clusters were identified. Heatmap values represent row-scaled Z-scores of Log_2_FC values for visualization only. Green and red colors indicate relative decreases and increases in expression, respectively, compared with the mean value of each gene across genotypes. The number of genes assigned to each cluster are indicated

First, we determined the differentially expressed genes (DEGs) between drought and control conditions for each line using a cut-off of FDR <0.05 and │Log_2_FC│≥1 (Supplementary Table S3). This analysis displays the transcriptional response to drought within each genotype. The comparison revealed that *alkbh10b-1* exhibited the highest number of DEGs (6205), followed by WT (5343) and OX-1 (4152) (Fig. 5B). Within each genotype the DEGs were comprised of a similar number of up- and down-regulated genes (Fig. 5B and C). We next examined all genes that met the significance threshold (FDR < 0.05) in all three genotypes in the drought versus control comparisons (Supplementary Table S4, all). From these, we selected those genes that displayed substantial differences in the drought-responsive expression patterns between the functional variants (Supplementary Table S4, selected genes). This selection identified 484 genes that exhibit a difference of at least 1 in Log₂FC (drought vs. control) between *alkbh10b-1* and OX-1. The selected genes were subsequently used for GO enrichment analyses separately for three classes categorized as biological process, cellular component, and molecular function (Fig. 6A–C). Remarkably, photosynthesis related processes emerged as the most strongly enriched term in all three classes, such as photosynthesis and light harvesting, within biological processes, photosystem I+II within cellular components, and chlorophyll and tetrapyrrole binding within molecular functions. Other terms within these classes relate to water transport and water channel activity but also responses to biotic and abiotic stress.

A cluster analysis of the selected genes identified four clusters with a number of genes ranging from 68 to 203 (Fig. 6D and Supplementary Table S4). Cluster 1 and 2 were defined by genes that in the *alkbh10b-1* mutant were differently expressed relative to both WT and OX-1 (Fig. 6D, right panel), while cluster 3 and 4 contain genes for which the expression in OX-1 differed from both WT and *alkbh10b-1* (Fig. 6D, left panel). To visualize these differences, row-scaled Z-scores are presented in a heatmap, with green and red colour indicating relative decreases and increases in expression, respectively. Cluster 1 comprised genes with enhanced up-regulation or attenuated down-regulation in *alkbh10b-1* compared to the other two lines (Table S5). Cluster 2 comprised genes with attenuated up-regulation or enhanced down-regulation in *alkbh10b-1* compared the other lines. Cluster 3 and 4 represent these respective patterns for OX-1. Within the selected gene set and under the applied filtering criteria, no genes showing significant counter-regulation between *alkbh10b-1* and OX-1 were identified. These clusters highlight genotype-specific transcriptomic signatures in response to drought, suggesting that *alkbh10b-1* and OX-1 utilize distinct sets of genes that may contribute to their differential stress tolerance.

We also analysed transcript levels under drought in *alkbh10b-1* and OX-1 relative to WT. In contrast to the drought-vs-control comparisons described above, this analysis was designed to identify genotype-specific differences in the expression levels of genes under the same drought treatment. In this comparison, only four genes showed a strong expression level difference between *alkbh10b-1* and WT at that specific state of water withholding (Fig. 5D and Supplementary Table S5). These include two genes with higher and two with lower expression levels in the mutant relative to WT, of which three were functionally uncharacterised and the fourth is *DERLIN-1*, encoding a protein that is part of the ERAD/ER protein quality-control machinery. In OX-1, no genes showed lower but 34 genes showed higher expression levels compared to WT under drought conditions, and most are functionally annotated (Fig. 5D and Supplementary Table S4). Of these, 14 genes encode mitochondrial proteins, most of them associated with the mitochondrial electron transport chain and oxidative phosphorylation (Fig. 5E and F). Although this is only a limited number of genes, their strong enrichment for mitochondrial functions suggests that coordinated regulation of this small gene set may provide an energetic advantage that helps to maintain cellular ATP homeostasis under drought, thereby contributing to the enhanced stress tolerance of OX-1.

### Overexpression of ***ALKBH10B*** preserves photosynthetic capacity under drought stress and modulates m^6^A levels of photosynthesis-related genes

**Fig. 7:**
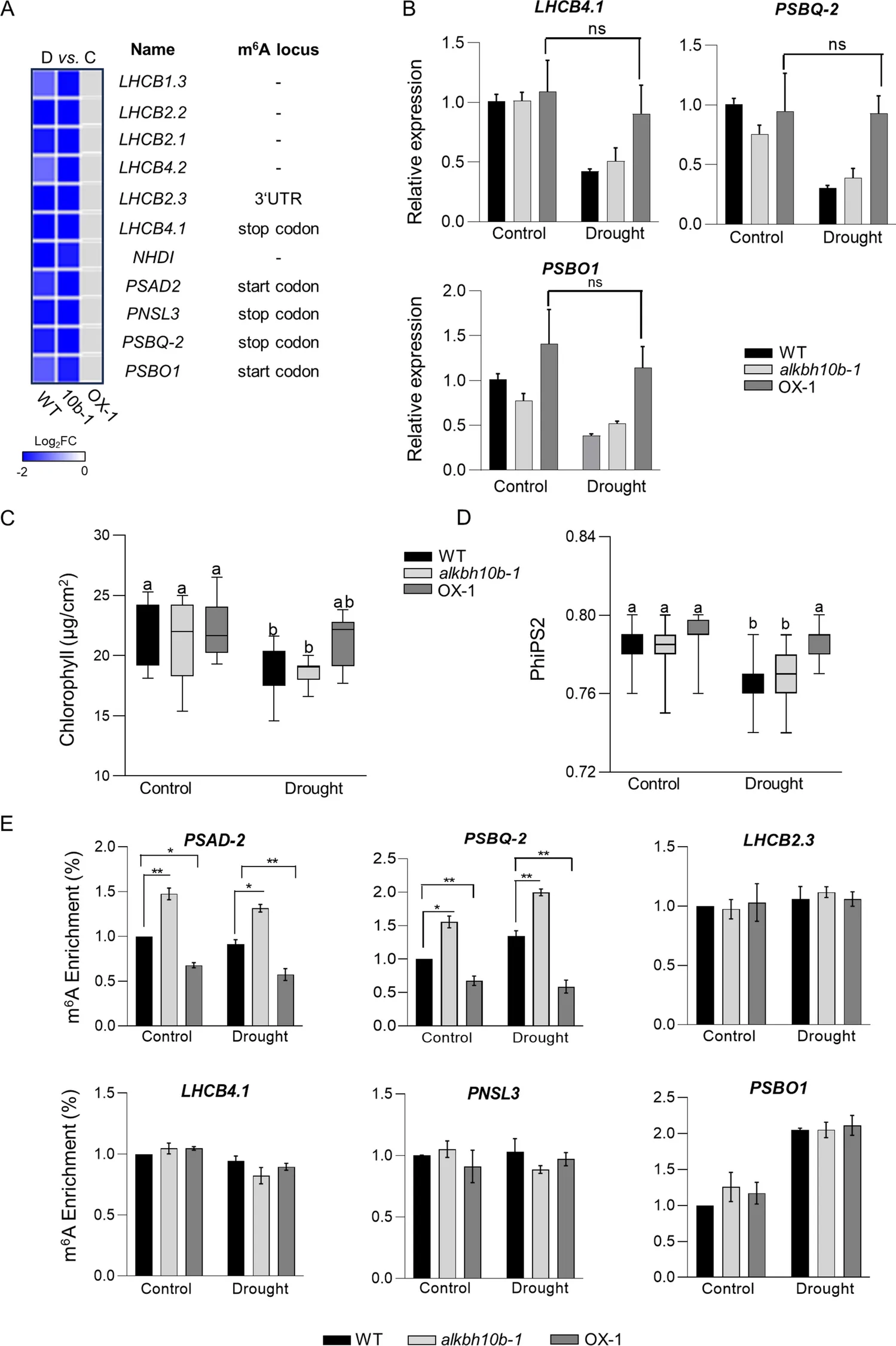
Effect of *ALKBH10B* on photosynthesis. **A** Heatmap representing the differential expression of photosynthesis-related genes in WT, *alkbh10b-1* and OX-1 under drought (D) *vs.* control (C) conditions determined by RNA-seq analysis. The blue squares in the heatmap indicate down-regulated genes, whereas grey squares represent no significant differences in expression. **B** Expression level of selected photosynthesis -related genes quantified by RT-qPCR under control and drought conditions. Values are the mean ± SE of three biological replicates (*n*=3). **C** Measurement of chlorophyll content and **D** Measurement of photosystem II activity under control and drought conditions. Values are the mean ± SE of *n*=10. Data were analysed through two-way ANOVA followed by Tukey’s multiple comparisons test (*P*<0.05) **E** m^6^A enrichment quantified using m^6^A-IP-qPCR under control and drought conditions. Values are the mean ± SE of three biological replicates (*n*=3). Asterisks indicate significant differences (Student’s t-test; *P < 0.05, **P < 0.01)

Since photosynthesis emerged as a prominent GO category, we had a closer look at the RNA-seq data of photosynthesis-related genes with differential expression in *alkbh10b-1* and OX-1 (Supplementary Table S4, selected genes). We found 14 genes related to photosynthesis all belonging to cluster 2 (Supplementary Table S4, Photosynthesis genes Cluster 2). The expression of these genes is generally down-regulated in all three genotypes with *alkbh10b-1* showing enhanced down-regulation, while OX-1 was the least affected. In addition, we identified a further set composed of 11 photosynthesis-genes that were significantly down-regulated in WT and *alkbh10b*−1 and which in OX-1 did not show significant differences (Supplementary Table S6). For three of these genes, LHCB4.1, PSBQ-2 and PSBO1, we validated the expression levels by RT-qPCR (Fig. 7B). Their expression patterns in WT and 10b-1 matched those observed in the RNA-seq data, and indeed no change in expression was observed for OX-1. In line with the transcript analysis, WT and the *alkbh10b-1* mutant showed a significant reduction in chlorophyll levels and PSII activity under drought stress compared to control conditions, whereas OX-1 plants were much less affected (Fig. 7C, D). To elucidate whether ALKBH10B directly affects the m^6^A modification of certain photosynthesis-related transcripts, we screened published m^6^A databases and identified potential m^6^A sites in *LHCB2.3*, *LHCB4.1*, *PSAD-2*, *PNSL3*, *PSBQ-2* and *PSBO1* (Fig. 7A). Quantification of m^6^A enrichment of these genes using m^6^A-IP-qPCR showed significantly decreased m^6^A levels only for *PSAD-2* and *PSBQ-2* in OX-1 plants compared to WT (Fig. 7E). Both genes had increased levels of m^6^A in the *alkbh10b-1* mutant in line with a stronger down-regulation in the expression, suggesting that these transcripts are putative targets of ALKBH10B-mediated demethylation. By contrast, the m^6^A levels of *LHCB2.3*, *LHCB4.1*, *PNSL3* and *PSBO1* remained largely unaffected, suggesting that they are not direct targets of ALKBH10B. These data indicate that rather than acting globally ALKBH10B selectively demethylates a subset of photosynthesis-related mRNAs, while other genes are indirectly affected.

### ALKBH10B modulates the expression and m^6^A enrichment of drought-responsive genes

**Fig. 8:**
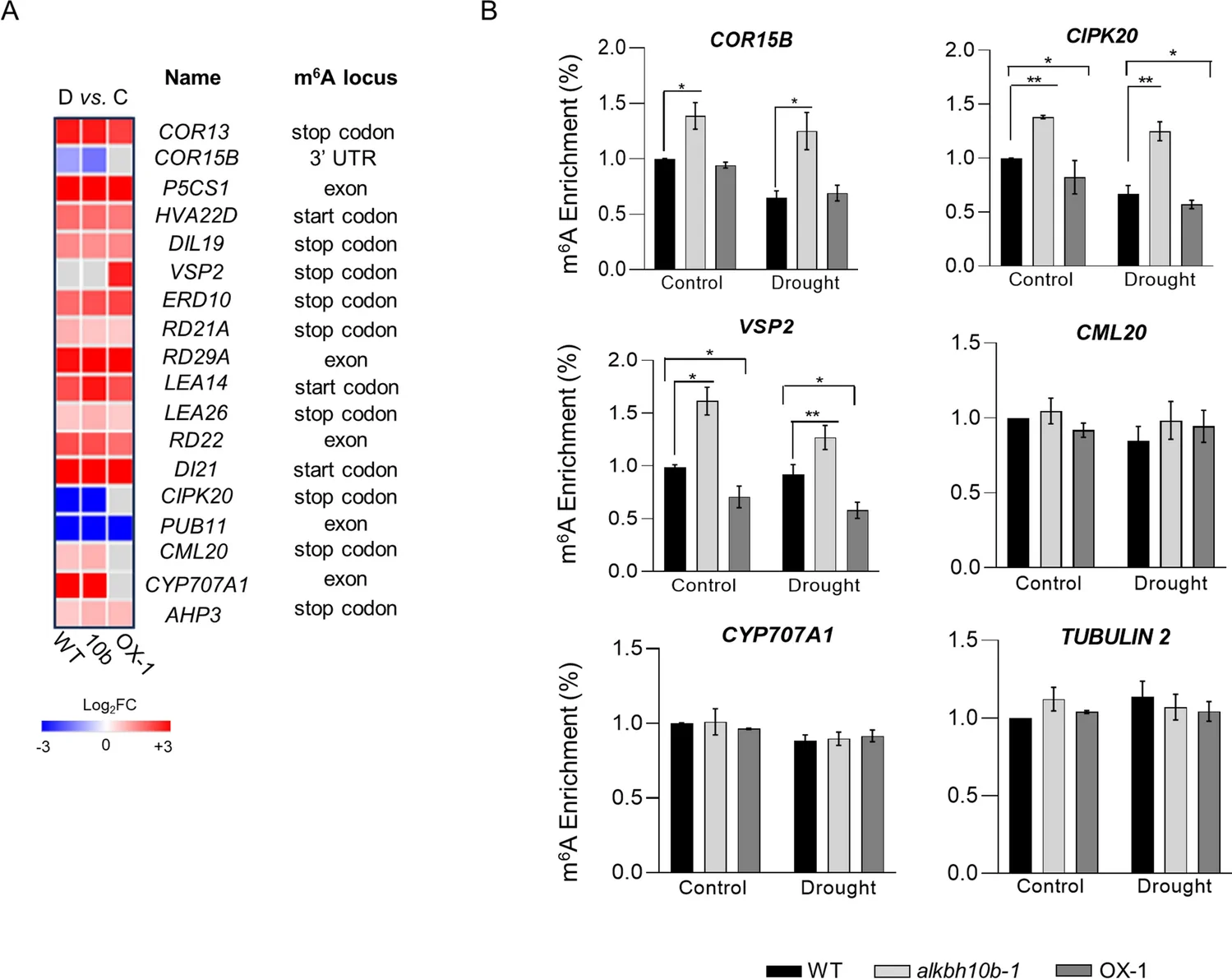
Effect of *ALKBH10B* on the regulation of known drought-related genes. **A** Heatmap representing the expression of drought-related genes in WT, *alkbh10b-1* and OX-1 under drought (D) *vs.* control (C) conditions. The red and blue squares in the heatmap indicate up- and down-regulated genes, respectively, whereas grey squares represent no significant differences in expression. **B** m^6^A level enrichment quantified using m^6^A IP-qPCR under control and drought conditions. *TUBULIN2* transcript was included as a non-m^6^A-modified negative control to demonstrate the specificity of the m^6^A-enrichment. Values are the mean ± SE of three biological replicates (*n*=3) and asterisks indicate significant differences between WT, *alkbh10b-1* mutant or OX-1 lines (Student’s t-test; *P < 0.05, **P < 0.01)

We also examined the effect of ALKBH10B on the regulation of known m^6^A modified drought related transcripts (Fig. 8A). Several of these genes can be classified as positive and negative drought regulators (Han et al. 2023), and our RNA-seq analysis demonstrated that most of these genes exhibited similar expression responses across all three genotypes. Several of these genes showed genotype-dependent differences (Fig. 8A), however, as observed before for the photosynthesis-related genes, some but not all of them also displayed changes in m^6^A enrichment (Fig. 8B). m^6^A levels of the positive regulators *COR15B*, *CIPK20* and *VSP2* were either decreased in OX-1, increased in *alkbh10b-1* or even both (Fig. 8B) with similar patterns observed under control and drought conditions, suggesting that these genes could be potential targets of ALKBH10B-dependent regulation. By contrast, m^6^A levels of two negative regulators, *CYP707A1* and *CML20*, remained unchanged in all lines, indicating that they are unlikely to represent primary targets of ALKBH10B-mediated m^6^A regulation under our experimental conditions.

To ensure the specificity of the m^6^A-IP-qPCR analysis, we included *TUBULIN2* as a negative control, as it is a widely recognized non-m^6^A-modified housekeeping gene. Our results confirmed that *TUBULIN2* showed no significant m^6^A-enrichment in any of the tested genotypes under drought or control conditions (Fig. 8B), thereby validating the specificity of m^6^A enrichment for our target transcripts.

## Discussion

In this study, our integrative approach combining transcriptomics, m⁶A profiling, physiological assays, and hormone quantification reveals a multi-layered mechanism in which the m^6^A demethylase ALKBH10B promotes drought stress resilience in Arabidopsis.

Our data show that drought stress globally triggers a coordinated transcriptional reprogramming of the whole m⁶A machinery in Arabidopsis including writers, readers, and erasers. While all components are up-regulated early after water withholding, *ALKBH10B* stands out due to its pronounced and sustained induction during prolonged drought (Fig. 1). This suggests that while the broader m^6^A machinery may initiate global epitranscriptomic adjustments, ALKBH10B specifically functions as a major responder during prolonged water deficit. This is in line with an elevated *ALKBH10B* expression shown previously under drought for sea buckthorn (Zhang et al. 2021) and tomato (Shen et al. 2023), where the plant prioritizes active m⁶A demethylation as part of its adaptive strategy. Since m⁶A demethylases have been implicated in fine-tuning stress-responsive transcripts (Shoaib et al. 2021; Han et al. 2023; Tang et al. 2021) this dynamic control likely enables the plant to rapidly modulate the stability and translation of stress-related mRNAs during water deficit. Whereas Han et al. (2023) established ALKBH10B as a positive regulator of drought tolerance, our study provides mechanistic insight into its contribution by linking ALKBH10B to hormone-mediated regulation of stomatal function, transcriptome-wide responses, and m⁶A-associated regulation of photosynthesis- and respiration-related pathways.

Characterization of plant lines with altered ALKBH10B content, revealed its role in modulating stomatal dynamics (Fig. 2), which is a critical factor governing water loss and drought resistance. Although differences in stomatal aperture between WT and OX-1 were moderate, the consistently reduced aperture in OX-1 seems sufficient to lower transpirational water loss during progressive drought periods. Despite the difference in stomatal apertures, ABA levels were similar between WT and OX-1 plants under control and drought conditions (Fig. 4), indicating that ABA abundance alone does not explain the observed stomatal phenotype. Accordingly, we also did not observe distinctive changes in the expression levels of ABA-dependent genes coding for proteins involved in stomata closure, such as *TGG1*, *SLAC1* or *KAT1* (Supplementary Table S3). Instead, reduced jasmonate accumulation in OX-1 may influence stomatal behavior under drought conditions (Fig. 4B and C). Although jasmonates are known to cross-talk extensively with ABA signalling in guard cells, the experimental evidence for their precise role in stomatal regulation is mixed. Depending on the system and conditions, jasmonates have been reported to both potentiate and antagonize ABA-induced stomatal closure (Munemasa et al. 2011). At present and based on our data, we cannot establish a direct causal hierarchy between ABA levels, jasmonate accumulation, and stomatal behavior; we, therefore, interpret these interactions as part of a complex hormonal network. It is also unclear how the reduced jasmonate content relates to ALKBH10B activity, since there is no clear correlation between jasmonate content and expression of genes related to jasmonate biosynthesis or catabolism (Supplementary Table S7). This suggests a post-transcriptional feedback mechanism, where ALKBH10B-mediated m^6^A demethylation likely alters translational efficiency of key proteins in these hormonal pathways, rather than their initial transcription.

What has become evident lately is that jasmonates can negatively modulate ABA-induced genes expression, as demonstrated for *RD29A/StRD29* in Arabidopsis and potato via external applications of ABA and MeJA (Bleker et al. 2024). In this experimental set-up, this is likely driven by enhanced JA-Ile content to which MeJA is converted intracellularly (Tamogami et al. 2012). Although the underlying molecular mechanism for this modulation of gene expression remains unresolved, we observed a comparable antagonistic interaction for *ALKBH10B* expression in this work (Fig. 3). Moreover, drought-induced expression of *ALKBH10B* was enhanced in the *jar1-11* mutant, highlighting the negative regulatory role of endogenous JA-Ile (Mahmud et al. 2022). Together, these findings indicate a complex integration of hormonal signals, where JA-Ile acts as a negative modulator that fine-tunes the magnitude of ABA-induced *ALKBH10B* expression. This antagonistic relationship suggests that the transcriptional control of *ALKBH10B* is specifically adapted to the hormonal signature of progressive drought. These results together suggest a regulatory loop where ABA and jasmonate appear to act primarily as upstream modulators of *ALKBH10B* expression, whereas ALKBH10B itself influences drought responses mainly through post-transcriptional fine-tuning of the hormonal landscape, thereby optimizing the drought response.

We did not observe any induction of *ALKBH10B* expression by MeJA itself, which seems to contradict results from Tang et al. (2021) showing that MeJA up-regulates *ALKBH10B* expression in seven-day-old seedlings. However, this apparent discrepancy with our findings can likely be attributed to differences in the developmental stage of the plants. Given that m^6^A dynamics vary across developmental stages (Zhang et al. 2024), ALKBH10B may participate in distinct jasmonate-responsive regulatory circuits during early seedling development versus prolonged drought stress in mature plants.

A similar context dependency may also apply to different stress conditions. The hormone-related role of ALKBH10B observed in our study differs from previous findings under salt stress. Under saline conditions, ALKBH10B was primarily associated with ABA sensitivity during seed germination and early seedling growth (Shoaib et al. 2021), whereas jasmonate-related effects were not a major focus. Our results from prolonged drought in mature rosette leaves indicate a stronger involvement of ABA-jasmonate interactions. These observations suggest that ALKBH10B may operate through both stress-specific and developmental stage-dependent signalling pathways, enabling flexible integration of distinct hormonal and environmental cues during stress adaptation.

Promoter analysis indicates that transcriptional regulation of ALKBH10B by ABA and jasmonate might be directly promoted via ABRE, MYB and MYC cis-elements (Fig. 3). The promoter of *ALKBH10B* also contains an AS-1 cis-regulatory element related to salicylic acid (SA); however, the expression of ALKBH10B was not notably affected by exogenous SA application in line with a lack of SA regulation shown for ALKBH10B in tomato leaves (Shen et al. 2023). Also, no significant differences in SA content were observed among all the genotypes under either drought and control growth conditions (Fig. S3), indicating that under our experimental conditions, i.e., age and tissue, ALKBH10B function is unlikely to be mediated through SA signalling.

Our physiological analyses suggest a likely role of m^6^A removal by ALKBH10B in hormone-dependent regulation of transpiration. However, our transcriptomic analysis reveals that ALKBH10B exerts effects beyond hormonal crosstalk. It is established that mitochondrial respiration plays a vital role in drought tolerance (Atkin and Macherel 2009) and our genotype-comparison under drought shows enhanced expression of a small but functionally coherent set of genes OX-1 plants compared to WT, nearly half of which are related to mitochondrial respiration and energy metabolism (Fig. 5D–F). This indicates an influence of m^6^A mRNA modification on this process. This is so far not investigated in plants, but m⁶A mRNA modification has recently been shown to regulate transcripts of genes coding for mitochondrial proteins in animal systems (Kahl et al. 2024). While this indicates an evolutionary conservation beyond the plant-animal divide, it gains another layer of complexity in plants due to the mitochondrial-chloroplast interplay in plant energy metabolism. Indeed, our transcriptional analysis showed that in the OX-1 plants certain photosynthesis-related genes remain stably expressed and PSII performance is maintained under drought, in contrast to the WT and *alkbh10b-1* mutant (Fig. 6 and 7). Similar findings have been observed for the ALTERNATIVE OXIDASE (AOX), where overexpression caused drought tolerance by maintaining not only mitochondrial but also chloroplast functions (Dahal and Vanlerberghe 2017).

In a recent study, it has been revealed that the m^6^A writer VIR regulates m^6^A modification of numerous photosynthesis-related transcripts during high light-induced photodamage, thereby regulating their gene expression at post-transcriptional level (Zhang et al. 2022). However, in our study, only the stable expression of *PSAD2* and *PSBQ-2* is consistent with the different m^6^A mRNA methylation patterns in the ALKBH10B variants and direct modulation remains to be experimentally demonstrated. Other photosynthesis-related transcripts showed no changes in m^6^A modification despite genotype-dependent changes in expression, likely reflecting indirect regulatory effects (Fig. 8). These effects may, for example, arise from demethylation of transcripts encoding transcriptional regulators, which could subsequently modulate broader photosynthesis-related gene networks. m^6^A modification has been observed for various transcription factors (Zhang et al. 2022) and we observed differential expression between WT and the function mutants for members of different transcription factor families (Supplementary Table S4 and S6).

ALKBH10B also regulates expression and m^6^A levels of certain drought-responsive transcripts, such as the kinase encoded by *CIPK20* (Fig. 8), which was shown to mediate stomata closure through the regulation of microtubule stability (Li et al. 2024). Sustained expression of *CIPK20* in OX-1 may contribute to reduced stomatal aperture compared to WT and *alkbh10b-1* (Fig. 2D). The function of these transcripts as well as their differential regulation and m^6^A mRNA modification by ALKBH10B are thus easily linked to the drought phenotype of the OX-1 and *alkbh10b-1* mutant lines. Nevertheless, as with the photosynthesis-related genes, not all expression changes correlate directly with m^6^A enrichment, reinforcing the likelihood of indirect downstream effects, such as post-transcriptional regulation, secondary signalling pathways, or downstream consequences of improved photosynthetic performance in OX-1 plants.

As noted in our transcriptomic analysis, all three genotypes underwent extensive transcriptional reprogramming in response to drought, with more than 4000 DEGs detected in each genotype (Fig. 5B). In contrast, direct comparisons of transcript levels between *alkbh10b-1* or OX-1 and WT under the same drought conditions revealed only a limited number of genotype-specific changes (Fig. 5D). This indicates that the core drought-responsive transcriptional program is largely shared among the three genotypes and suggests that ALKBH10B does not act as a primary driver of the transcriptional stress response. Instead, its biological significance likely resides in the epitranscriptomic fine-tuning of specific target transcripts who subsequently influence downstream physiological processes. This observation is consistent with the established role of m⁶A demethylases, which primarily regulate RNA fate at the post-transcriptional level rather than acting as major transcriptional regulators. Consequently, modulation of a relatively small number of key target transcripts can produce substantial physiological effects and phenotypic differences. This is supported by the fact that even minor shifts in the m^6^A status of key transcripts, such as those involved in photosynthesis or hormone signalling, can lead to the significant physiological phenotypes observed under drought stress without requiring a massive alteration of the total transcriptome. These results emphasize that even minor, targeted changes in m^6^A modification are sufficient to drive significant drought adaptation, highlighting the precision and subtlety of ALKBH10B’s regulatory role.

Interestingly, it was shown very recently that the m^6^A writer, MTA, also imparts drought tolerance through m^6^A-dependent and -independent impacts on mRNA regulation (Ganguly et al. 2025). Similar to VIR mentioned above, MTA appears to act more globally than ALKBH10B. Moreover, whereas VIR primarily influences photosynthesis-related genes and MTA affects drought-responsive transcripts, ALKBH10B impacts both processes as well as mitochondrial respiration. Importantly, the observed limited overlap between m^6^A-modified transcripts and differentially expressed genes underscores the need of distinguishing direct epitranscriptomic regulation, where m^6^A affects directly target transcripts, from secondary transcriptional reprogramming that arise as downstream consequence of altered m^6^Alevels. This distinction is critical for interpreting epitranscriptomic datasets and will require future integration of m⁶A epitranscriptomic profiling with targeted analyses of stress-related pathway components including proteomic studies. Recognizing this distinction is critical for accurately interpreting how specific epitranscriptomic modifications contribute to broad physiological adaptations.

## Supplementary Information

Below is the link to the electronic supplementary material.

Supplementary file1 (PDF 484 KB)

Supplementary file2 (XLSX 12 KB)

Supplementary file3 (XLSX 3098 KB)

Supplementary file4 (XLSX 522 KB)

Supplementary file5 (XLSX 21 KB)

Supplementary file6 (XLSX 21 KB)

Supplementary file7 (XLSX 19 KB)

## Data Availability

Raw RNA seq data used in this study are available under (PRJNA1357100) at SRA repository from NCBI.
