# Supplementary material for: ALKBH10B mediated epitranscriptomic regulation enhances drought tolerance of Arabidopsis thaliana via hormonal cross-talk and sustained photosynthesis
Source: Plant Cell Rep. 2026 Aug 21;45(9):257. doi: 10.1007/s00299-026-03951-1 (PMC13498519; doi:10.1007/s00299-026-03951-1)
Supplement: Supplementary file 1 [file 299_2026_3951_MOESM1_ESM.pdf]

ALKBH10B mediated epitranscriptomic regulation enhances drought tolerance of *Arabidopsis thaliana* via hormonal cross-talk and sustained photosynthesis

Yasira Shoaib[1], Rongpeng Han[2], Sabarna Bhattacharyya[1], Alexandra Finkenauer[1], Katharina Gutbrod[3], Peter Dörmann[3], Hunseung Kang[2*], Ute C. Vothknecht[1*], and Fatima Chigri[1]

[1] Institute for Cellular and Molecular Botany (IZMB), University of Bonn, Kirschallee 1, 53115 Bonn, Germany
[2] Department of Applied Biology, College of Agriculture and Life Sciences, Chonnam National University, Gwangju 61186, Korea
[3] Institute of Molecular Physiology and Biotechnology of Plants (IMBIO), University of Bonn, Karlrobert-Kreiten-Straße 13, 53115, Bonn, Germany

***Correspondence:**

Ute C. Vothknecht, vothknecht@uni-bonn.de, Orcid_ID 0000-0002-8930-0127

Hunseung Kang, hskang@chonnam.ac.kr

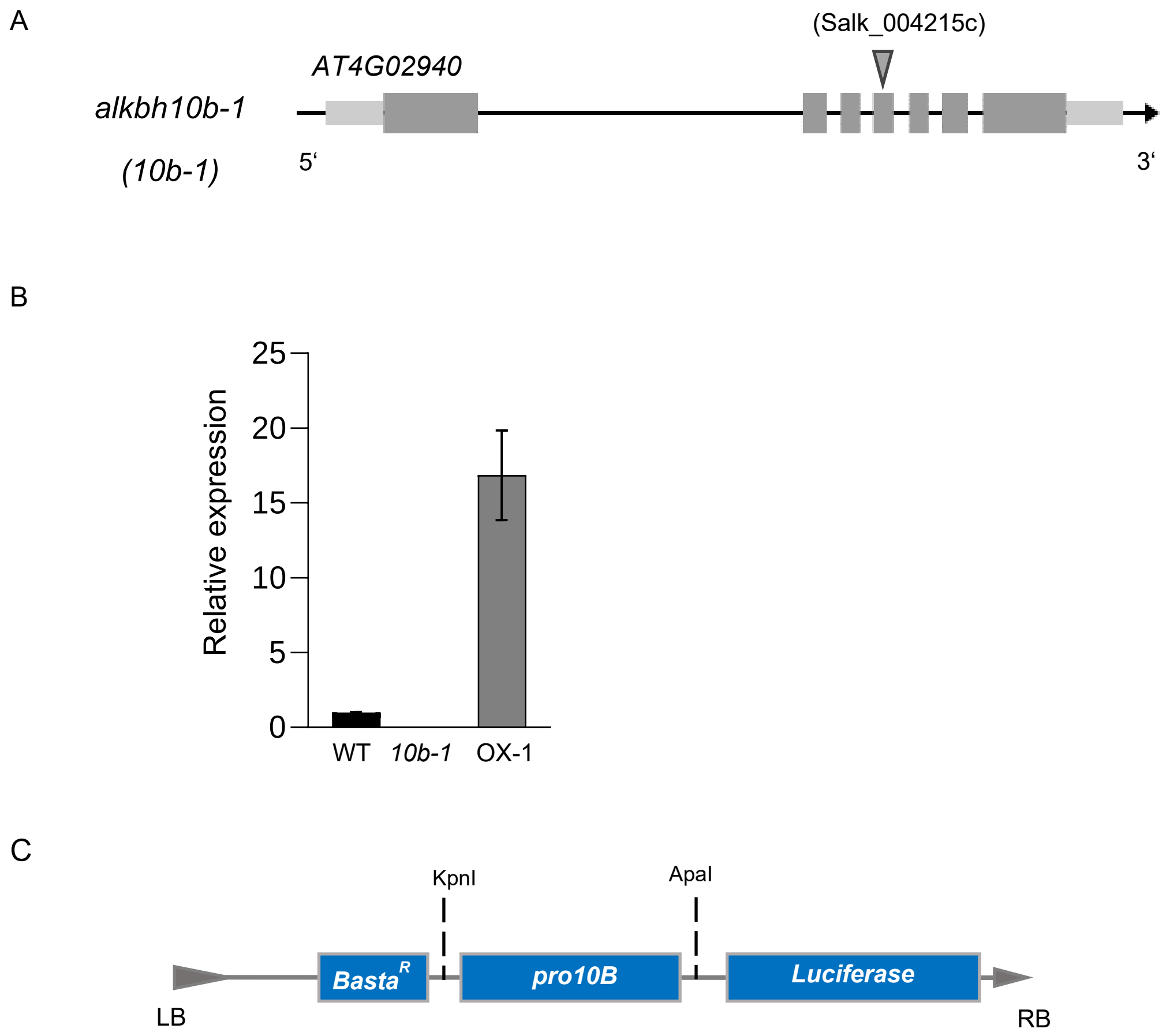

Fig. S1 Characterisation of *ALKBH10B* transgenic lines and cloning of a p*ALKBH10B*::FLUC construct. A Schematic representation of the T-DNA insertion in the *ALKBH10B* mutant (Salk_004215c) **B** Transcript abundance in *alkbh10b-1* null mutant and 35S:ALKBH10B overexpression lines (OX-1) using RT-qPCR. **C** Schematic representation of the *pALKBH10B::FLUC* cassette in the LB: Left border; RB: Right border. *pro10B:* 1000 bps upstream of the start codon of *ALKBH10B*

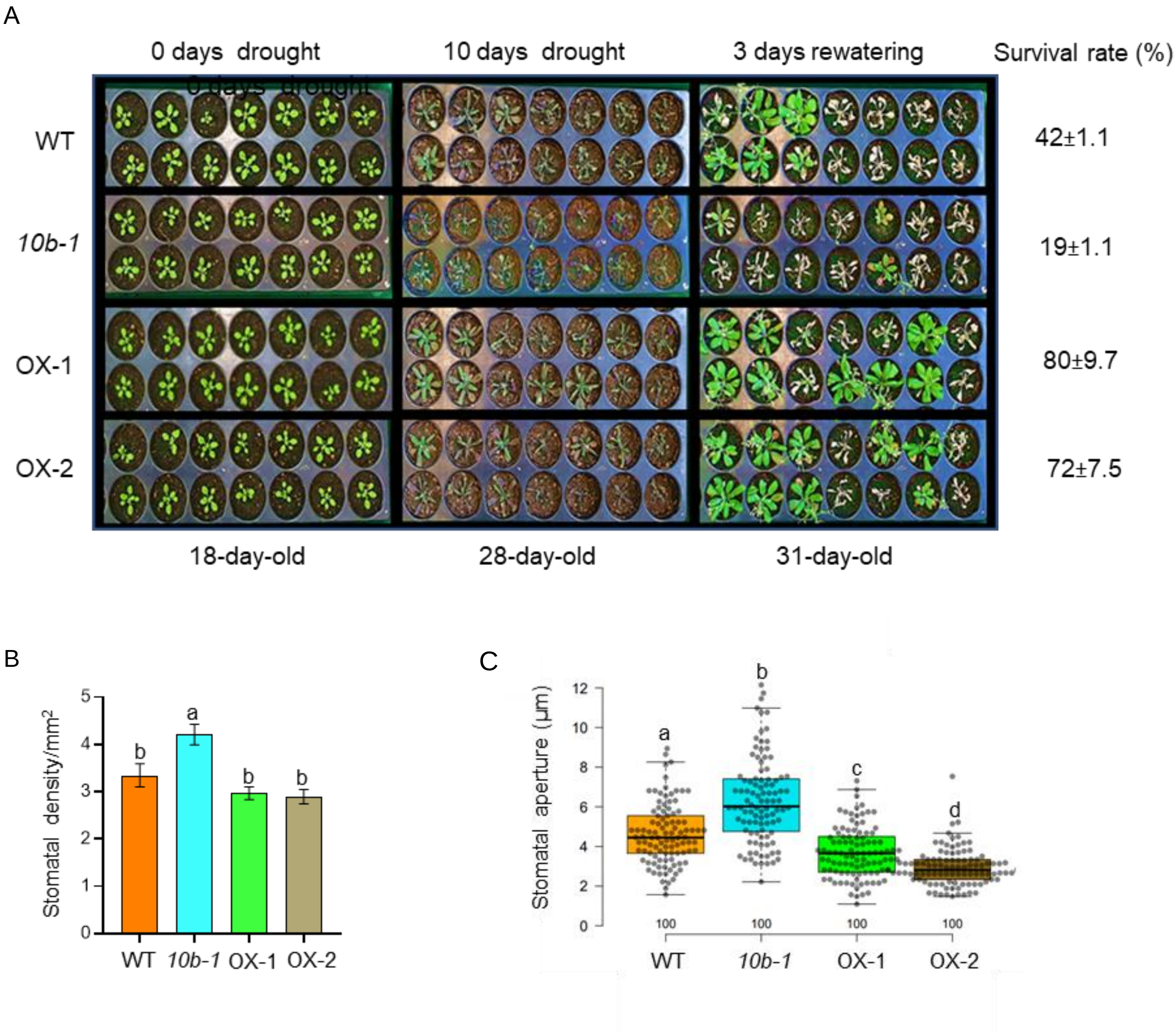

Fig. S2 Phenotyping of WT, *alkbh10b-1,* and two independent lines of 35S:ALKBH10B (OX-1 and OX-2) under drought stress. A Drought stress was applied to 18-day-old plants by with-holding water for 10 days and survival rate was measured after 3 days of rewatering. **B** Stomatal density and **c** aperture measured in 21-days old plants under control conditions. Values are the means ± S.E of three biological replicates each consisting of two technical replicates. Data were analysed through one-way analysis of variance (ANOVA) ($P<0.05$), followed by Multiple comparison analysis (Tukey‘s honest significant difference [HSD test]).

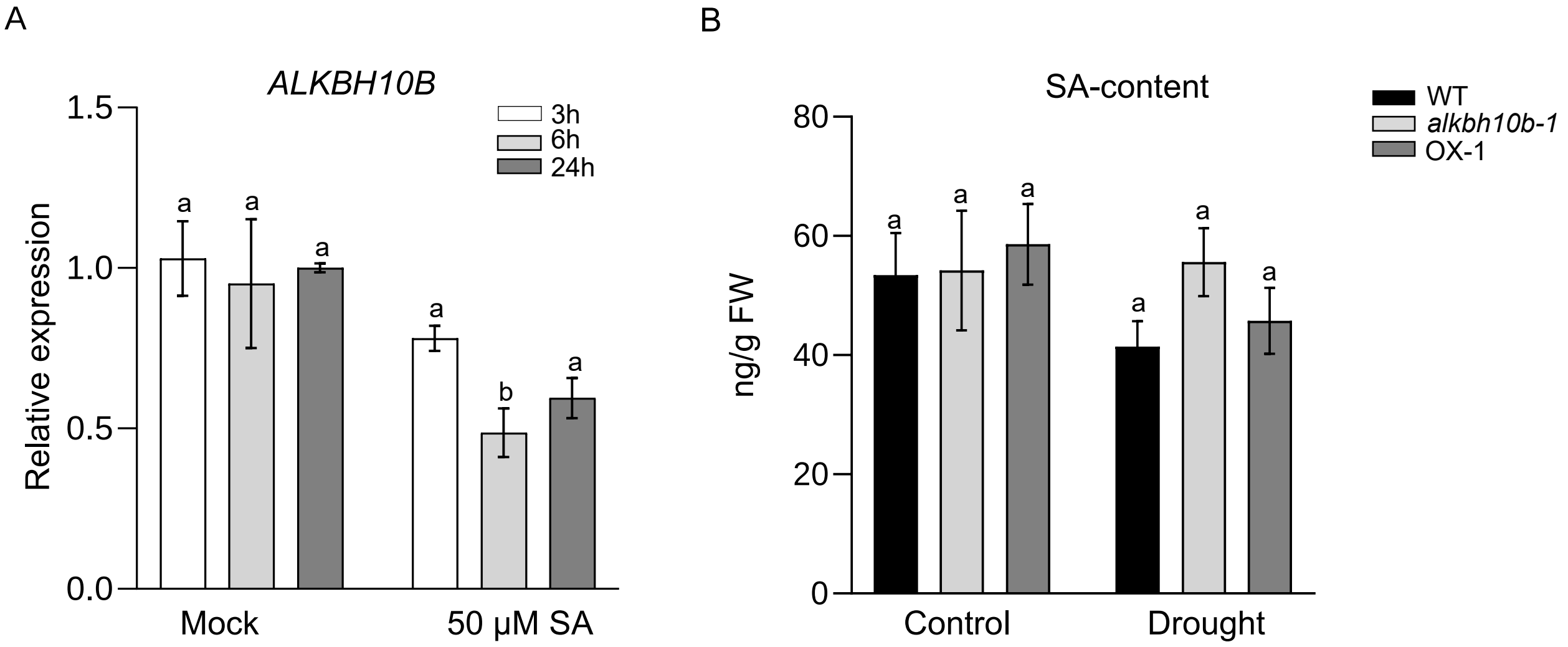

Fig. S3 Effect of salicylic acid (SA) on the expression of *ALKBH10B* and analysis of its content in WT, *alkbh10b-1* and OX-1. A Relative expression of *ALKBH10B* in WT plants grown on ½ MS plates for 21 days and treated with either water (mock) or 50 µM SA for 3, 6 and 24 hours quantified through RT-qPCR. Data represent mean ± SE of 3 biological replicates (n=3). **B** Analysis of SA levels in WT, *alkbh10b-1* and OX-1 grown under control and drought stress conditions. Data represent mean ± SE of 5 biological replicates (n=5) each containing pooled extracts from 2 plants. The data were analysed through two-way Anova followed by Tukey Post-Hoc HSD test (P< 0.05)

Table S2. Summary of total reads and aligned reads in RNA seq samples under control and drought conditions. For each treatment, three biological replicates were performed and each contained RNA pooled from two individual plants**.** After quality control, reads were aligned against the TAIR 10 Arabidopsis genome assembly.

Samples	Replicates	Total reads	Aligned reads	Aligned reads (%)
WT C	1	9651540	8160377	84.55
	2	10239533	8941160	87.32
	3	10152705	8852143	87.19
alkbh10b-1 C	1	9851158	8618778	87.49
	2	8323243	7722258	84.93
	3	9067730	7911594	87.25
OX-1 C	1	9562982	8284411	86.63
	2	8627557	7429189	86.11
	3	10828200	9645760	89.08
WT D	1	10159554	8678291	85.42
	2	9144232	7872269	86.09
	3	10813648	9018582	83.40
alkbh10b-1 D	1	10469305	9008836	86.05
	2	11204970	9710227	86.66
	3	8218264	7009357	85.29
OX-1 D	1	9147531	7876938	86.11
	2	8747080	7514616	85.91
	3	10023597	87065493	86.85
